# Synthesis and physical characteristics of narrow bandgap chalcogenide SnZrSe _3_

**DOI:** 10.12688/openreseurope.15168.1

**Published:** 2022-12-13

**Authors:** Rokas Kondrotas, Remigijus Juškėnas, Arūnas Krotkus, Vidas Pakštas, Artūras Suchodolskis, Algirdas Mekys, Marius Franckevičius, Martynas Talaikis, Katri Muska, Xiaofeng Li, Marit Kauk-Kuusik, Victor Kravtsov

**Affiliations:** 1 Center for Physical Sciences and Technology, Vilnius, 10257, Lithuania; 2 Institute of Photonics and Nanotechnology, Vilnius University, Vilnius, 10257, Lithuania; 3 Department of Materials and Environmental Technology, Tallinn University of Technology, Tallinn, 19086, Estonia; 4 Institute of Applied Physics, Chisinau, MD2028, Moldova

**Keywords:** ABX3 chalcogenides, crystal structure, optoelectronic properties, bandgap

## Abstract

**Background:** The development of organic/inorganic metal halide perovskites has seen unprecedent growth since their first recognition for applications in optoelectronic devices. However, their thermodynamic stability and toxicity remains a challenge considering wide-scale deployment in the future. This spurred an interest in search of perovskite-inspired materials which are expected to retain the advantageous material characteristics of halide perovskites, but with high thermodynamic stability and composed of earth-abundant and low toxicity elements. ABX _3_ chalcogenides (A, B=metals, X=Se, S) have been identified as potential class of materials meeting the aforementioned criteria.

**Methods:** In this work, we focus on studying tin zirconium selenide (SnZrSe _3_) relevant physical properties with an aim to evaluate its prospects for application in optoelectronics. SnZrSe _3_ powder and monocrystals were synthesized via solid state reaction in 600 – 750 °C temperature range. Crystalline structure was determined using single crystal and powder X-ray diffraction methods. The bandgap was estimated from diffused reflectance measurements on powder samples and electrical properties of crystals were analysed from temperature dependent *I-V* measurements.

**Results: **We found that SnZrSe _3_ crystals have a needle-like structure (space group – *Pnma*) with following unit cell parameters: a=9.5862(4) Å, b=3.84427(10) Å, c=14.3959(5) Å. The origin of the low symmetry crystalline structure was associated with stereochemical active electron lone pair of Sn cation. Estimated bandgap was around 1.15 eV which was higher than measured previously and predicted theoretically. Additionally, it was found that resistivity and conductivity type depended on the compound chemical composition.

**Conclusions:** Absorption edge in the infrared region and bipolar dopability makes SnZrSe _3_ an interesting material candidate for application in earth-abundant and non-toxic single/multi-junction solar cells or other infrared based optoelectronic devices.

## Plain language summary

The sun provides an enormous amount of energy to the planet and has the potential to meet all humanity power demands. Photovoltaic (PV) technologies, i.e. devices that convert sunlight directly to electricity, play an essential role in harvesting this solar energy. While the sun will shine and provide clean energy for many million years to come, the manufacture of PV technologies uses finite earth resources and depending on the type of technology can be energy demanding and therefore emit large amounts of CO _2_. For these reasons, scientists are constantly improving synthesis and fabrication processes to increase material utilization, improving device structure to increase power conversion coefficient and study stability dynamics to prolong device lifetime. In parallel, new materials composed of earth-abundant and non-toxic chemical elements are explored which could potentially replace or complement current technologies. In this work, we focus on investigating a promising compound composed of non-toxic and earth-abundant elements from ABX _3_ chalcogenide family materials – SnZrSe _3_. Using a solid state reaction method, we synthesized SnZrSe _3_ crystals and measured their optical and electrical properties. We found that a critical semiconductor feature – bandgap (which indicates what wavelength light will be absorbed) of SnZrSe _3_ is in the suitable range for application in solar cells. Additionally, electrical measurements indicated that SnZrSe _3_ can be engineered to behave as n- or p-type semiconductor which is very important for the formation of semiconductor devices. In summary, we believe that SnZrSe _3_ is an interesting candidate for development of greener PV technologies.

## Introduction

Perovskite class of materials describe compounds sharing the same type of crystal structure (perovskite) and have specific stoichiometry – ABX _3_, where A and B are cations, and X is an anion. The unprecedented development of organic/inorganic lead halide perovskites (HP) for application in light-emitting diodes ^1^, X-ray detectors ^2^ and photovoltaics ^3^ has been witnessed in the last decade. Despite their tremendous success, poor thermodynamic stability under ambient conditions ^4^ and the presence of a toxic chemical element (Pb) ^5^ question their wide-scale deployment in the future. This spurred an interest in search of materials with the same advantageous physical properties as HP, but with much higher chemical stability and environmentally benign compositions ^6^.

The success of HP is thought to be caused by an unusual material property – defect tolerance. This means that despite having a high density of crystallographic defects in the semiconductor, charge carrier lifetime on the order of µs can be readily obtained ^7^. However, there is no consensus on the origins of the defect tolerance and it has been associated with multiple material aspects that are characteristic to metal HP, such as: special bonding-antibonding character when antibonding states are at the top of the valence band ^8^, coordination environment and ionic nature of chemical bonds ^6, 9^, and with a high static dielectric constant effectively screening charged defects ^6, 10^.

When searching for perovskite-inspired materials it is therefore important to know how defect tolerance is related to the structural characteristics of the compound. For instance, it has been proposed that materials comprised of cations with lone electron pairs can potentially have defect tolerance characteristics ^10^. Alternatively, a more straightforward way is to explore compounds with perovskite structure and ABX _3_ composition. For this reason, and because of high thermodynamic stability, low toxicity and earth-abundant chemical composition, chalcogenide perovskites (X=Se, S) have gained a lot of interest in recent years ^11^.

The first study on chalcogenide perovskite properties using first-principles calculations was introduced by Sun *et al.* ^12^ (Note: that the study was limited to chalcogenide composition A ^2+^B ^4+^X ^2-^_3_, where A=Ca, Sr, Ba; B=Ti, Zr; Hf, X=Se, S). It was shown that chalcogenide perovskites had photovoltaic-relevant properties and some even exceeded well-established materials used in solar cells, showing their great potential in photovoltaics. This sparked an interest in chalcogenide perovskites, of which BaZrS _3_ has drawn the most attention ^13– 16^. It was, however, shown that among many ABX _3_ chalcogenides, under normal conditions, only very few exist as perovskite structures ^17^: BaZrS _3_, BaHfS _3_, SrZrS _3_, SrHfS _3_, CaZrS _3_. All of them are wide bandgap (> 1.7 eV) semiconductors, and therefore have a relatively narrow application range in optoelectronics. However, other ABX _3_ chalcogenides although having a non-perovskite ground phase, are also stable compounds, have bandgaps in the infrared region and have high optical absorption.

In this work, we focus on examining SnZrSe _3_ which is predicted to have a narrow ~ 0.65 eV bandgap ^18^. SnZrSe _3_ is composed of low-toxicity and earth-abundant chemical elements and is stable under ambient conditions. Additionally, SnZrSe _3_ and SnZrS _3_ are both stable in the needle-like phase suggesting a full chemical range of miscibility in SnZrSe _x_S _3-x_ alloy and therefore a high tuneability of the bandgap. Experimentally estimated bandgaps for SnZrSe _3_ and SnZrS _3_ were 0.86 eV and 1.2 – 1.4 eV, respectively, however, the data are available only from one source ^19^. Therefore, herein we aim to synthesize and estimate the optical and electrical properties of SnZrSe _3_ for potential application in infrared-based optoelectronic devices.

SnZrSe _3_ was synthesized by solid state reaction in a powder form and as monocrystals. SnZrSe _3_ was found to crystallize in orthorhombic structure with space group *Pnma* as confirmed by single-crystal and powder diffraction methods. The absorption edge was estimated at around 1.15 eV indicating to the narrow bandgap nature of SnZrSe _3_, but considerably higher than reported before and predicted theoretically. Finally, depending on the chemical composition, SnZrSe _3_ was found to behave as an *n*- or *p*-type semiconductor highlighting bipolar dopability in SnZrSe _3_ compound.

## Methods

SnZrSe _3_ samples were synthesized via a solid state reaction method. Elemental precursors comprising Sn (99.995%, AlfaAesar, -100 mesh), Zr (99.5% STREM Chemicals, -50 mesh), Se (99.999%, AlfaAesar, -200 mesh) and SnI _2_ (99.99%, SigmaAldrich, -10 mesh) were weighted in an Ar filled glove box. The total mass of the precursor before annealing was 0.5±0.005 g plus 0.01±0.005 g of SnI _2_. Then, the precursors were introduced to a quartz ampoule (inner diameter – 8 mm, outer – 10 mm, length – 150 mm) which was capped to protect the precursors from the ambient environment when taken outside the glovebox. Before sealing, all 13 ampoules were degassed for 30 – 60 min under approximately 2 Pa pressure. Four ampoules were immersed in an ultrasonic bath to remove precursors stuck to the quartz walls. Ampoules were sealed under vacuum using a flame from an oxygen and propane gas mixture and the final ampoule length was in the 60 – 80 mm range. First batch of ampoules had a carbonized inner wall to avoid quartz reaction with precursor materials during the synthesis step. However, we did not observe any traces of chemical reactions of precursors with a quartz ampoule and the carbonization step was not used for the rest of the samples.

Ampoules were introduced in the tube furnace, 10 of them in a horizontal and 3 of them in a vertical (placed in ceramic holder) positions at the centre of the heating zone. The heating process included two steps: (i) temperature was raised to the top temperature within 3 – 4 h which was varied in the 600 – 800 °C range and held for 10 – 80 h; (ii) in the second step, the temperature was reduced to 400 – 600 °C within 8 – 60 h, and furnace power was switched off leaving to cool down naturally for a few hours. We found that larger crystals were formed when the temperature was reduced slowly in the second stage, instead of maintaining a high furnace temperature for a prolonged period of time.

Single crystal X-ray data of SnZrSe _3_ were obtained at 20 °C using an Xcalibur E diffractometer equipped with an Eos CCD space detector (Agilent Technologies) and a monochromatic source of MoK _α_ radiation (graphite monochromator, Oxford inst.). The data were collected and processed using the program CrysAlisPro (Oxford Diffraction Ltd., Version 1.171.37.35, provided with diffractometer) and were corrected for the Lorentz and polarization effects, and absorption. All calculations to solve the structures and to refine the proposed models were carried out with the SHELXS97 ^20^ and SHELXL2014 software packages ^21^ ( SHELX is free of charge for academic users).

The structure was refined by the full matrix least squares method on F2 with anisotropic displacement parameters using the program SHELXL ^20^. Crystallographic data and structure refinement details and geometric parameters are given in CIF file (see *Underlying data* ^22^). CIF files were deposited with the Cambridge Crystallographic Data Centre CCDC/ICSD, deposition number CSD 2166561, and can be accessed upon request ( https://www.ccdc.cam.ac.uk/).

Powder diffraction was measured using Rigaku diffractometer SmartLab with 9 kW rotating Cu anode in Bragg-Brentano geometry. Before measurements, crystals collected from the ampoule were grinded in an agate mortar. Diffractograms were recorded in the range of 10 to 60 2θ degrees with a scan step of 0.01 degree using linear D/tex ultra detector.

Raman measurements were performed using an inVia Raman microscope (Renishaw, Wotton-under Edge, UK) equipped with the 1800 lines/mm grating and thermoelectrically cooled (-70 °C) CCD camera at 532 nm wavelength excitation. Raman spectrum was taken using a 50x/0.75 NA (Leica) objective lens. Laser power was restricted to 0.05 mW and the integration time was set to 300 s. The Raman frequencies were calibrated using the silicon standard according to the line at 520.7 cm ^-1^.

Diffuse reflectance was measured using Shimadzu UV-3600 two-beam spectrometer equipped with a multi-purpose compartment MPC-3100. Samples were placed in a 60 mm integrating sphere and a barium sulphate target was used for calibration.

Scanning Electron Microscope (SEM) images were taken using Helios Nanolab 650 equipped with a field emission gun. Chemical composition was recorded with an energy dispersive spectrometer (Oxford inst.) embedded in SEM. Before Energy Dispersive X-ray (EDX) measurements were made, calibration using a Cu plate was performed and other parameters such as working distance (5 mm), accelerating voltage (20 kV) and exposure time (30s) were kept constant.

Temperature dependent current-voltage measurements were recorded using a Keithley 6487 picoammeter/voltage source. The sample was placed in a closed helium cycle cryostat (Janis CCS-100/204) and the temperature was controlled via a digital temperature controller. Current-voltage measurements were made from 295 to 100 K with a 10 K temperature step size. Once the set temperature was reached on the thermocouple, it was waited for 3 min for temperature to equalize on the sample.

All data visualization and processing were performed using OriginPro 2021 version 9.8.0.200 ( https://www.originlab.com/index.aspx?go=Products/Origin) and files are accessible in *Underlying data* ^22^. Alternatively, data can be treated and analysed using freely available software Labplot ( https://labplot.kde.org/). Visualization of crystalline structure was done using freely available software Mercury ( https://www.ccdc.cam.ac.uk/solutions/csd-core/components/mercury/).

## Results

### SnZrSe _3_ solid state synthesis

**Figure 1.**
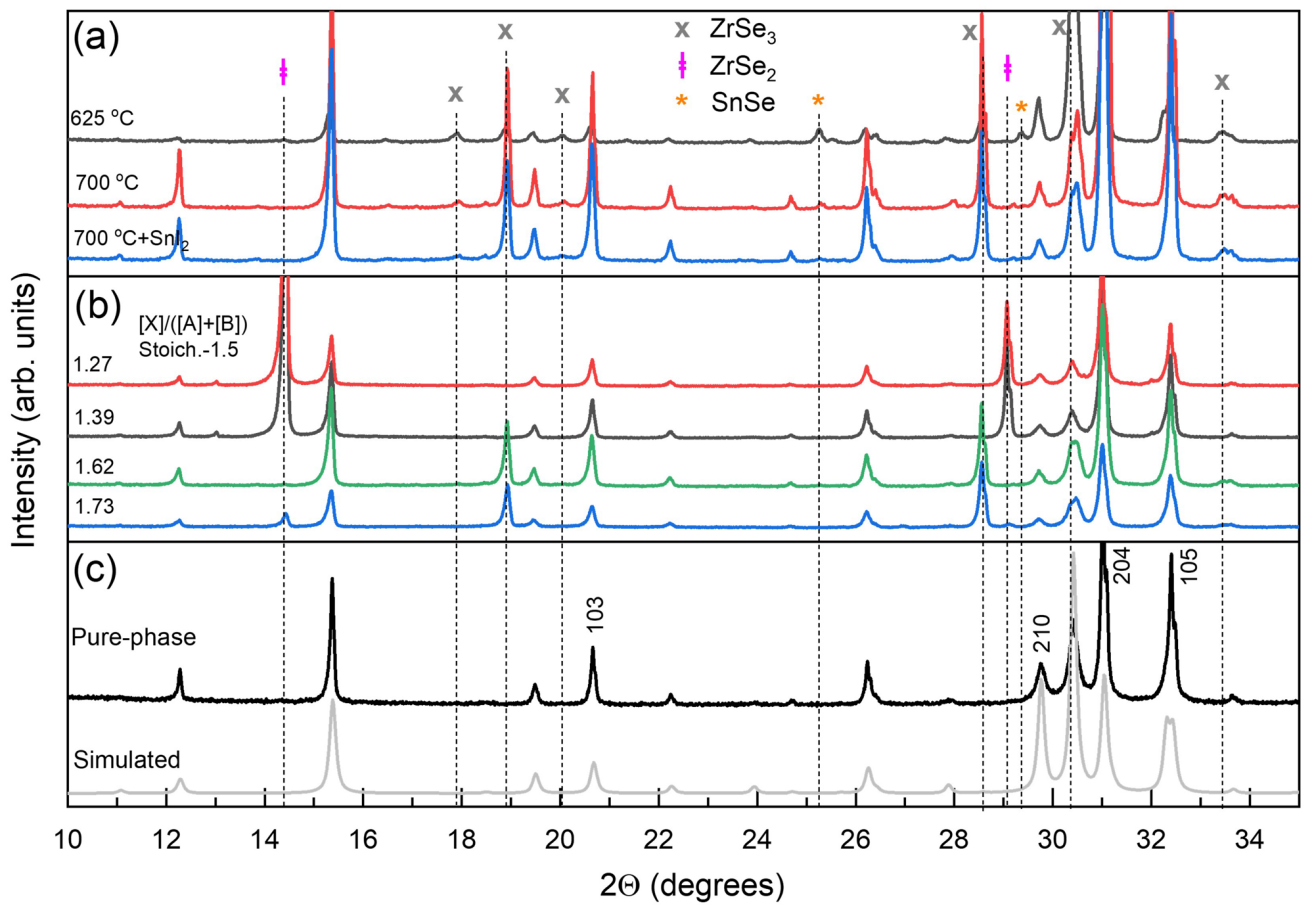
Phase composition analysis determined by X-ray diffraction method. ( **a**) Diffractograms of SnZrSe _3_ powder synthesized at various temperatures in a vertical position without and with transport agent. ( **b**) Diffractograms of SnZrSe _3_ powder samples synthesized in the same experiment at 740 °C temperature in a horizontal position with different initial Se-content in the precursor as indicated in the graph. ( **c**) XRD pattern of collected and grinded crystals synthesized at 700 °C in horizontal position with a transport agent. Diffractogram of pure SnZrSe _3_ phase was simulated using CIF file ( *underlying data* ^22^).

SnZrSe _3_ was synthesized via solid state reaction from precursors in elemental form. In most cases, a transport agent (SnI _2_) was used to accelerate the reaction and grow single crystals. In other cases, no additives were used. However, despite testing various temperature profiles, changing the ampoule orientation or adding/removing transport agent, we were not able to obtain pure single-phase powder. Various shapes, sizes and forms of crystals were always present inside an ampoule after annealing. Regardless of the synthesis conditions used, SnZrSe _3_ was formed in addition to one or two of the following binary phases that were identified in diffractograms as follows: ZrSe _3_ (ICDD# 03-065-2351), ZrSe _2_ (ICDD# 04-005-5128) and SnSe (ICDD# 04-009-2257). Based on multiple trials, we noticed that the following experimental parameters had a deciding factor on the phase composition: (i) spatial separation of precursor materials and (ii) Se overpressure in the ampoule. When the ampoule was oriented vertically in the annealing zone, ZrSe _3_ and traces of SnSe phases were present despite the annealing temperature applied ( Figure 1a). Interestingly, the addition of transport agent in a form of SnI _2_ did not change the phase composition. This suggested that when precursors were in close proximity, the solid state reaction was not accelerated or favoured for the formation of SnZrSe _3_ in the presence of a transport agent. In cases when ampoule was oriented horizontally (powder was spread over a certain length), phase composition strongly depended on the Se content. Using Se-deficient precursor, a large amount of ZrSe _2_ and absence of ZrSe _3_ phases were observed in the powder ( Figure 1b). Once stoichiometric or Se-rich precursors were used, little to no amount of ZrSe _2_ was found, which indicated that the formation of ZrSe _3_ phase was highly favoured under Se-rich atmosphere. Note that when the ampoule was oriented horizontally no secondary Sn-related phases were observed.

Based on the obtained results we propose a simplified reaction mechanism described below. During the temperature ramp in the synthesis process, it is suggested that SnSe is formed first, which crystallizes already at 300 °C ^23^:


$$Sn(l)+\frac{1}{x}Se_{x}(g)\to SnSe(s)\;(1)$$

where *l*, *g* and *s* indicate liquid, gaseous and solid states of matter; *x* denotes Se molecule size, which can be from 2 to 8 depending on the temperature ^24^. In parallel, Zr reacts with Se vapour and ZrSe _2_ is formed as the most stable form of Zr – Se system ^25, 26^:

$$Zr\;(s)+\frac{1}{x}Se_{x}(g)\to ZrSe_{2}(s)\;(2)$$

Above 600 °C, SnSe enters a gaseous phase and starts to react with zirconium diselenide:

$$SnSe\;(g)+ZrSe_{2}(s)\to SnZrSe_{3}\;(s)\;(3)$$

When there is a surplus of Se in the atmosphere, a competing reaction occurs:

$$ZrSe_{2}(s)+\frac{1}{x}Se_{x}(g)\to ZrSe_{3}(s)\;(4)$$

Therefore, we believe that the thermodynamic balance between reactions (3) and (4) which depends on spatial separation of materials, partial Se pressure and annealing temperature determine the final product composition. While inspecting the ampoule annealed sequentially first at 700 °C and then at 800 °C, it was noticed that after second annealing large SnZrSe _3_ crystals deteriorated, and small fine needle-like crystals typical for ZrSe _3_ were found (Figure S1 in *Extended data* ^22^). This indicated that SnZrSe _3_ decomposes at higher temperatures. Therefore, an optimal temperature window for the synthesis of SnZrSe _3_ lies in the 600 – 750 °C temperature range.

To obtain a single-phase SnZrSe _3_ powder, a part of large needle-shaped crystals was collected from the ampoule’s walls and was grinded in a mortar. Other crystals were used as-grown for single crystal X-ray diffraction (XRD), Raman scattering and electrical measurements. The XRD pattern of thus obtained powder is presented in Figure 1c. A very good match was found between the experimental XRD pattern and the one simulated from single crystal XRD data. No secondary phases were detected. Note that intensity of XRD peaks of (h0l) planes was higher than in the simulated pattern ( Figure 1c). This shows that a preferred orientation of (h0l) planes was present even in the powder sample because needle-like shaped grains tend to orient with their long axis parallel to the surface. Single-phase powder was later used for optical measurements.

### Determination of the SnZrSe _3_ structure

To determine the crystalline structure of SnZrSe _3_, needle shaped crystal with dimensions of 0.25 × 0.02 × 0.015 mm and synthesized at 700 °C was selected for single crystal X-ray diffraction measurements. The summary of crystallographic information is presented in Table 1. More details on the refinement procedure and parameters can be found in the experimental description and crystallographic information file ( *Underlying data* ^22^) attached to the publication.

**Table 1.** Summary of SnZrSe _3_ single crystal crystallographic information. Crystallographic information was collected using single crystal XRD method and based on SnZrSe _3_ crystal synthesised at 700 °C temperature. The key information was selected.

Compound	Crystal system	Space group	Unit cell parameters		
			a, Å	b, Å	c, Å
SnZrSe _3_	Orthorhombic	*Pnma*	9.5862(4)	3.84427(10)	14.3959(5)
**Atomic coordinates**					
Atom	WP	x	y	x	U(eq)
Sn(1)	4c	4556(1)	7500	6664(1)	22(1)
Zr(1)	4c	1608(1)	2500	5498(1)	12(1)
Se(1)	4c	2697(1)	2500	7191(1)	16(1)
Se(2)	4c	3318(1)	7500	4876(1)	13(1)
Se(3)	4c	-155(1)	7500	6104(1)	11(1)
**Geometric parameters**					
Within ribbon (along *b*)		Intra ribbon (along *a*)		Inter ribbon (along *c*)	
Bond	Length, Å	Bond	Length, Å	Bond	Length, Å
Sn(1)-Se(1)	2.7291(5)	Se(2)-Se(2)	3.771	Sn(1)-Se(3)	3.225
Sn(1)-Se(2)	2.8352(7)	Sn(1)-Se(2)	3.573	Se(1)-Se(3)	3.736
Zr(1)-Se(1)	2.6514(7)				
Zr(1)-Se(2)	2.6798(5)				
Zr(1)-Se(3)	2.7041(5)				

**Figure 2.**
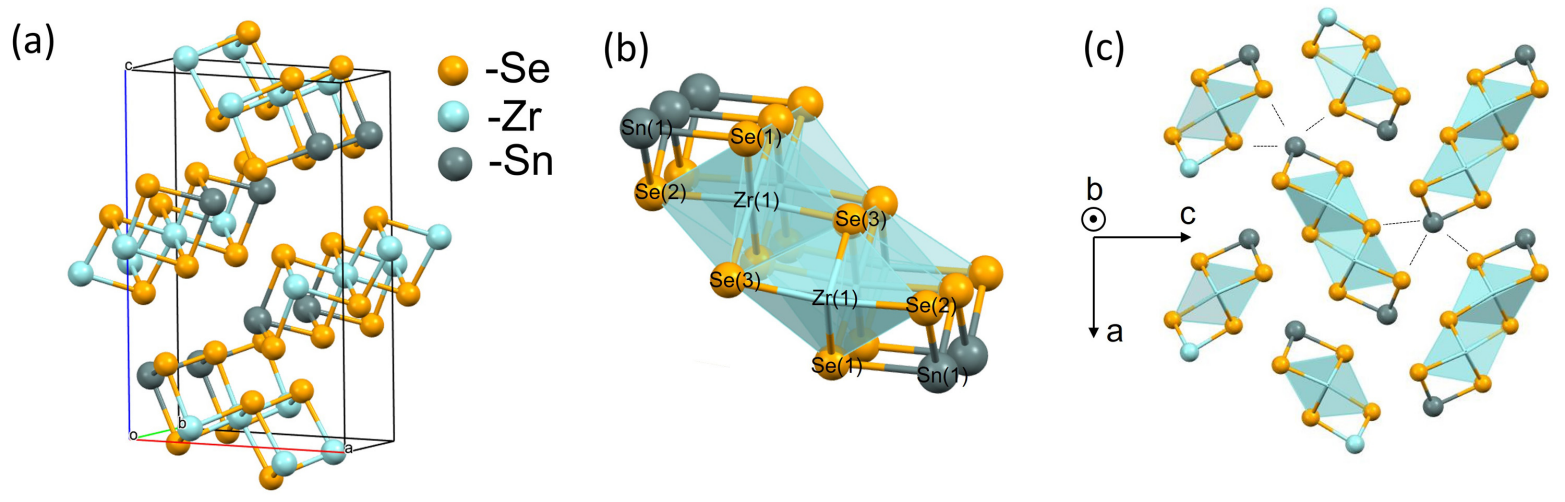
Crystalline structure of needle-like SnZrSe _3_ phase. Single crystal XRD results confirmed that SnZrSe _3_ crystal is orthorhombic ( **a**) The main building block of SnZrSe _3_ structure is ribbon which is presented in ( **b**) with labelled atom sites. ( **c**) The projection of SnZrSe _3_ crystal structure on ( *a*, *c*) plane highlight the anisotropy around Sn cation. Dashed lines indicate > 3 Å interatomic distances in Sn coordination environment.

It was determined that SnZrSe _3_ crystal is orthorhombic and belongs to space group *Pnma* ( Figure 2a). The calculated unit cell parameters were *a* = 9.5862(4) Å, *b* = 3.84427(10) Å, *c* = 14.3959(5) Å and unit cell volume V = 530.52(3) Å ^3^. The needle axis of the crystal habit corresponded to the shortest crystallographic axis *b*. The X-ray structural analysis showed that the crystalline structure of SnZrSe _3_ compound was isomorphous and isostructural to the sister compound SnZrS _3_^27^. The fundamental building block of the crystalline structure was a ribbon ( Figure 2b) which comprised of double edge-sharing Zr octahedra extending along *b* direction indefinitely. Within a ribbon and along *b* direction atoms are held by strong bonds as evidenced by the short interatomic distances of < 3 Å ( Table 1). On the contrary, ribbons themselves are held together via van der Waals forces because of the longer interatomic distances (> 3 Å) found along directions *a* and *c* ( Table 1). Within a unit cell cations occupy two non-equivalent sites Sn(1) and Zr(1), whereas anions have three distinct positions ( Figure 2b). Sn(1) is coordinated with three Se atoms forming a trigonal pyramidal geometry, whereas Zr is coordinated with six Se atoms forming a distorted octahedra geometry. Additionally, the bonding environment around Sn cation in terms of bond length was found to be anisotropic ( Figure 2c). Sn-Se bonds which are shorter than 3 Å are part of the ribbon structure, whereas in other directions they are longer than 3 Å giving a clear spatial separation between ribbons.

### Optical characterisation of bandgap

**Figure 3.**
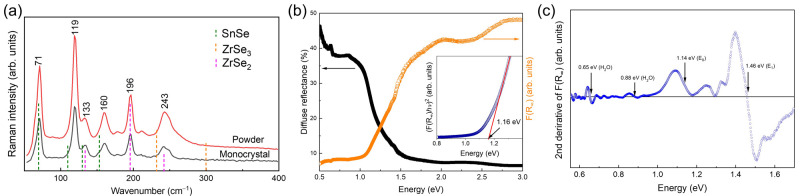
Raman spectra and optical properties of SnZrSe _3_ powder. ( **a**) Raman scattering spectra of single phase SnZrSe _3_ powder and monocrystal synthesized at 700 °C. Excitation wavelength - 532 nm. ( **b**) Diffuse reflectance spectrum and thereof calculated apparent absorption using eq. 5 of single-phase SnZrSe _3_ powder. Inset – Tauc plot of SnZrSe _3_ around absorption edge. (c) Second derivative of measured apparent absorption as a function of energy of single-phase SnZrSe _3_ powder.

For the optical characterization of SnZrSe _3_, single-phase powder as determined from XRD measurements was selected. To examine if there were any impurity phases present on the surface of the powder, Raman scattering measurements were conducted. As seen before, XRD results showed no evidence of secondary phases ( Figure 1c). For the Raman scattering study, additional samples were measured – a SnZrSe _3_ monocrystal which served as a reference case. Raman spectra contained vibrational bands located at 71, 119, 133, 160, 196 and 243 cm ^-1^ ( Figure 3a). Because there is no reference Raman spectrum of SnZrSe _3_ in the literature, we first examined if there were secondary phases that had been observed in XRD patterns. Positions of the main and the most intensive Raman bands of ZrSe _2_^28^, SnSe ^29^ and ZrSe _3_^30^ are shown as dotted lines ( Figure 3a). No evidence of ZrSe _3_ phase was found. However, one Raman band of SnSe (71 cm ^-1^) and three bands of ZrSe _2_ (134 cm ^-1^, 196 cm ^-1^, 242 cm ^-1^) overlap very well with some of the SnZrSe _3_ bands. Despite a good match, we believe it is not a response from secondary phases, but because of structural similarities between SnZrSe _3_ and ZrSe _2_ (SnSe) that give rise to similar vibrational bands. The main structural element in ZrSe _2_ is ZrX _6_ octahedral which is also the case in SnZrSe _3_ ( Figure 2b). Coordination environment of Sn in SnSe and SnZrSe _3_ also share similar structural features therefore rendering alike Raman bands. In addition, the Raman spectrum of SnZrSe _3_ monocrystal perfectly matched with Raman spectrum of the SnZrSe _3_ powder sample. This strongly supports that SnZrSe _3_ powder was free of secondary phases and overlapping bands were a result of similar structural characteristics between SnZrSe _3_ and ZrSe _2_ (SnSe).

To estimate the absorption and to calculate optical bandgap and other critical points in the electronic band, diffuse reflectance was measured of the single-phase powder sample ( Figure 3b). According to the Schuster-Kubelka-Munk formulation, apparent absorption and diffuse reflection are related as follows ^31^:

$$F(R_{\infty })=\frac{{\left(1-R_{\infty }\right)}^{2}}{2R_{\infty }}=\frac{K}{S}\;(5)$$

Where, R _∞_- diffuse reflectance, K – apparent absorption, S – reflection coefficient.

Assuming S did not change considerably over the measured energy range, F(R _∞_) was taken to reflect the SnZrSe _3_ absorption coefficient. Diffused reflectance together with the calculated F(R _∞_) are depicted in Figure 3b. To estimate the bandgap, we first applied the common Tauc method ^32^. In brief, material’s absorption coefficient (for photon energy above the bandgap) is proportional to the material’s bandgap as follows: α *E _ph_* ≈C( *E _ph_*- *E _g_*) ^n^, where α is absorption coefficient, C – constant reflecting joint density of states in the bands, *E _ph_* – is a photon energy, *E _g_* – material’s bandgap and *n* -exponent depending on the optical transition nature: for direct transition *n*=2, for indirect *n*=0.5. Then, to find the bandgap value, ( *αE _ph_*) ^*n*^ is plotted versus *E _ph_* and fitted linearly. A linear fitting region is extrapolated until it crosses x axis where α≈0 and the crossing point is defined as material’s bandgap. Direct transition was considered for SnZrSe _3_ therefore (F(R _∞_)· *E*) ^2^ vs *E* plot was used ( Figure 3b, inset). The fitted linear region near the absorption edge resulted in a bandgap value of around 1.16 eV. In the second approach, to estimate the position of critical points (CP) in the band structure, the second derivative of F(R _∞_) was calculated ( Figure 3c). To reduce background noise and highlight CP features, data points were smoothed using Savitzky-Golay methods with 3 ^rd^ order polynomial and a 100 point window. Note, that we did not fit the derivative with the Aspnes’ function, because to obtain correct fitting results, high-accuracy measurements of material dielectric function are required. Nevertheless, the position of CP can be still estimated as the inflexion point of the CP feature, which typically has one positive and one negative extrema ( Figure 3c, indicated by arrows). In addition, to validate the certainty of CPs identified in the spectrum, we calculated second derivative under various smoothing conditions for the same sample and measured the diffuse reflectance on SnZrSe _3_ sample made from another batch. In all cases, inflexion points were located at the same positions (Figure S2 in *Extended data* ^22^). First, clear CP features below the SnZrSe _3_ absorption edge were observed at 0.65 and 0.87 eV, respectively. The position of these CPs was in very close agreement with H _2_O absorption bands which are located at 1940 nm (~0.64 eV) and 1450 nm (~0.85 eV) ^33^. This indicated that H _2_O was present in BaSO _4_ which was used as a white reference plate in the diffuse reflectance measurements. Such a case is quite common when BaSO _4_ is used as a reference plate. Other CPs were located at 1.14 and 1.46 eV ( Figure 3c). The low energy CP was assigned to the SnZrSe _3_ bandgap because it coincided well with the value estimated from the Tauc plot ( Figure 3b). However, all other higher energy CPs cannot be assigned to a specific optical transition and is beyond the scope of this work. Based on the diffuse reflectance results, SnZrSe _3_ bandgap was around 1.15 eV which is almost twice as large as predicted from first-principles ^18^ and is substantially higher than measured by Richard ^19^. First-principles calculations are known to underestimate the bandgap whereas the value measured by Richard was very close to the water absorption band located at 1450 nm. Although we consistently found a bandgap value of about 1.15 eV, more samples such as thin films, and other bandgap measurement methods would be useful to consolidate the real bandgap value of SnZrSe _3_ and nature of transition (direct/indirect).

### Electrical properties

**Figure 4.**
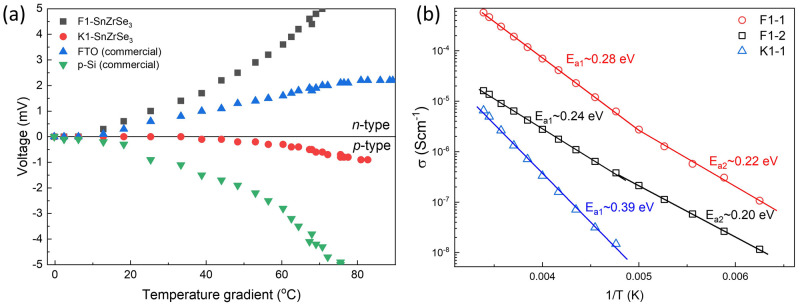
Electrical properties of SnZrSe _3_ crystals ( **a**) Voltage as a function of a temperature difference between probes measured on SnZrSe _3_ crystals using hot-point-probe method. Commercial FTO (fluorine doped SnO _2_) and boron doped Si wafer were used to calibrated system. ( **b**) Electrical conductivity as a function of reciprocal temperature in three single-crystal SnZrSe _3_ samples.

Conductivity type is a very important factor when considering the formation of semiconductor heterojunctions. Without intentional doping, conductivity usually depends on the dominating intrinsic point defects in the material, which in turn are related to the compound stoichiometry. To study conductivity properties of SnZrSe _3_, we first tested the largest needle-like crystals with a hot-point-probe method. This method allows us to identify the conductivity type by observing current/voltage sign upon increase in temperature gradient between probes. Before measurements, the system was calibrated with well-known *n*-type commercial fluorine doped SnO _2_ (SigmaAldrich) sample and boron doped commercial *p*-type (100) Si wafer. It turned out that some as-grown crystals showed a constant positive voltage change upon temperature gradient increase and some – negative ( Figure 4a). This indicated that SnZrSe _3_ can exhibit both *n* and *p*-type conductivity. Note that *n*-type behaviour was much more pronounced suggesting higher carrier concentration was present in *n*-type than in *p*-type crystals or much higher mobility of electrons. This was also in-line with calculated resistivity which was two orders of magnitude higher for *p*-type sample ( Table 2). To find if there was a relation between off-stoichiometry and conductivity type, we measured the chemical composition of crystals showing *n* and *p*-type behaviour, respectively. The average composition of the crystals measured over more than 5 points are summarized in Table 2. In both samples, the cationic ratio A/B was identical, whereas the *n*-type crystal was slightly more Se-rich. At this point, it is difficult to confirm if the different Se quantity was the origin of respective conductivity type and would require more samples to be synthesized and tested. In addition, there was quite a large compositional variation as seen from the high standard deviation. Important to note that since iodine was used as a transport agent to facilitate the solid state reaction during synthesis, it could be inadvertently introduced in the lattice giving rise to extrinsic doping. Iodine could not be detected by EDX, but we acknowledge that very small amounts (beyond the EDX detection limit) can have a significant contribution to the conductivity behaviour. In summary, although the origin of doping is not clear in SnZrSe _3_, the bipolar conductivity behaviour is a very desirable feature for semiconductor-based technologies ^34^.

**Table 2.** Chemical composition and electrical parameters of SnZrSe _3_ crystals from F1 and K1 batches. Two batches (F1 and K1) of crystals were studied which showed pronounced *n*- and *p*-type conductivity behaviour. Chemical composition clearly showed correlation with composition in uniform crystal samples.

Sample	Sn, at.%	Zr, at.%	Se, at.%	Sn/Zr	Se/(Sn+Zr)	ρ _s_, Ω·cm	E _a1_, eV	E _a2_, eV
*n*-F1 (HHP)	21.5±1.1	20.9±0.4	57.6±1.3	1.03	1.36			
*p*-K1 (HHP)	22.1±1.5	21.4±0.9	56.5±1.0	1.03	1.30			
F1-1	22.0±0.12	21.0±0.13	57.0±0.13	1.05	1.33	1.5·10 ^3^	0.28	0.22
F1-2	21.0±0.52	21.5±0.12	57.5±0.41	0.98	1.35	3.9·10 ^4^	0.24	0.20
K1-1	19.4±0.09	22.4±0.2	58.2±0.14	0.86	1.39	1.7·10 ^5^	0.39	-

To estimate the ionization potential (E _a_) of defects contributing to the conductivity, temperature dependent *I-V* curves were measured. To avoid grain boundary effects, as monolithic as possible SnZrSe _3_ samples were selected (Figure S3 in *Extended data* ^22^). However, due to their small size, their conductivity type could not be measured directly by HPP and was assumed from the measurements of larger crystals from the same batch. We found that the resistivity of samples varied in 10 ^3^ – 10 ^5^ Ω·cm range highlighting the insulating nature of SnZrSe _3_. Calculated E _a_ of defects are summarized in Table 2 and Figure 4b. We see that for F1 series samples two E _a_ were calculated, but because conductivity is the product of carrier concentration and carrier mobility, it cannot be ruled out that reduction in E _a_ was because of the change of carrier scattering mechanisms, and therefore increased mobility upon temperature decrease. Overall, all defects that were found to contribute to the conductivity can be considered as deep defects, because E _a_ » 0.025 eV, which is a thermal excitation energy at room temperature (kT), k is Boltzmann constant and T – temperature (295 K). Chemical composition of the crystals was also measured ( Table 2) to link composition with conductivity behaviour. These crystals were much more homogenous as evidenced by a small standard deviation. Notably, the specific resistance was found to correlate with the Sn/Zr ratio: the more Sn-rich sample was, the smaller resistivity. In addition, it is likely that Sn-rich composition also led to *n*-type conductivity, whereas Sn-deficiency – to the *p*-type. This would also explain why in the Sn-deficient sample (K1-1) we observed very different E _a_ than in the other two cases. E _a_ in F1 samples was related to the donor defects, whereas in K1 – to the acceptor type defects. This was not obvious when the composition of large crystals was measured because of their chemical inhomogeneity. Resistivity can also be related to the gradual change of Se content as well. However, to rely on the Se ratio to the cations is less accurate, because we found small amounts of oxygen present in the crystals, especially if exposed for a long time in the ambient environment. The oxygen is most likely adsorbed from the air or in some amorphous state because phase composition determined by XRD of the powder sample that was stored for more than half a year under ambient conditions (T=20-25 °C, RH=30-60%) did not change (Figure S4 in *Extended data* ^22^). This also shows high thermodynamic stability of the SnZrSe _3_.

## Discussion

A characteristic structural feature of perovskite compounds is that cation B forms an octahedra geometry with sharing corners ^35^. In fact, the stability of perovskite structures are predicted by estimating Goldschmidt’s tolerance factor, *t*, which reflects an ability to squeeze octahedra in a cubic sub-lattice ^36^. However, ABX _3_ chalcogenides do not follow predictions based on *t* only. None of the ABX _3_ chalcogenides exist in a perfect cubic structure even when *t*=1.0 (which is a golden ratio for cubic perovskite). Because chalcogen ionic radius is much larger than that of oxygen, the octahedral factor ( *µ*) must also be accounted for. Then, *t* is plotted against *µ*, it can be clearly seen that only a few ABX _3_ chalcogenides fall in the region of having a perovskite structure ^17^. For SnZrSe _3_ calculated *t* and *µ* values are 0.79 and 0.36, which falls outside the perovskite region ( *t* > 0.85, *µ* > 0.4).

Instead, ABX _3_ chalcogenides are found to exist in distorted perovskite (model structure – GdFeO _3_), needle-like (model structure NH _4_CdCl _3_) and hexagonal (model structure BaNiO _3_) structures. Other crystal structures also exist in ABX _3_ chalcogenides, for example, CeTmS _3_-type in A ^3+^B ^3+^X ^2-^_3_^37^, CuTaS _3_-type in A ^1+^B ^5+^X ^2-^_3_^38^ and there are other possible variations although more rare. In needle-like and hexagonal structures, B cation octahedra is sharing edges instead of corners as is the case in SnZrSe _3_ ( Figure 2c). This will give rise to anisotropic carrier transport because low effective mass is expected in the direction of edge-sharing octahedra compared to other directions. Indeed, high anisotropy in electronic band dispersion was shown in SnZrS _3_ and SnZrSe _3_ (needle-like phase) using the first-principles calculations ^18^.

Additionally, it is important to note the difference in bonding environment between SnZrSe _3_ and other needle-like ABX _3_ chalcogenides containing alkali or alkaline earth cations, for instance, SrZrSe _3_ or RbCdCl _3_. In SrZrSe _3_, cation A is positioned almost equidistantly from the nearest neighbouring atoms ^39^, whereas in SnZrSe _3_ as shown before there is a clear anisotropy in terms of interatomic distances ( Figure 2c). The origin of the ribbon-like structure in SnZrSe _3_ could be related to the stereochemically active lone electron pair. On theoretical grounds, it has been shown that stereochemically active lone pairs lead to distorted low symmetry crystal structures ^40^. Sn in SnZrSe _3_ is in a +2 valence state which leads to two unpaired 5s electrons. For the binary compounds, if there is a strong interaction between cation s states and anion p states, electronic stabilization is achieved through lattice distortion and lone electron pair is ejected outwards forming a structural void. That leads to asymmetric bonding around lone electron pair containing cation. Many chalcogenides containing cation with lone electron pair such as Sb _2_Se _3_, Sb _2_S _3_, Bi _2_S _3_, SnHfS _3_, SnZrS _3_, PbHfS _3_ and PbZrS _3_ have a ribbon-like low symmetry crystal structure. Because of these structural similarities at least binary compounds also share some electronic and optical characteristics, for instance indirect bandgap with a small difference between direct and indirect gaps and anisotropic carrier transport properties ^41^. Based on first-principles calculations, SnZrSe _3_ is predicted to have a small (< 0.1 eV) difference between indirect and direct gaps as well ^18^. Such characteristic is highly desired in absorber materials for photovoltaics because high absorption coefficient and long carrier lifetime can be realised simultaneously ^41^.

In this work, we found that SnZrSe _3_ showed *p* as well as *n* type conductivity behaviour. This is in contrast to other multicomponent well-known photovoltaic materials such as Cu(In,Ga)Se _2_, Cu _2_ZnSn(Se,S) _4_, CuSb(Se,S) _2_ and Cu _2_Sn(Se,S) _3_ where usually one type carrier is dominant. Because of low formation energy of Cu vacancy defect (acceptor type), these materials are intrinsically p-type ^42– 45^. Inability to alter the conductivity type and magnitude on demand, puts constrains on the device structure and requires formation of heterojunctions. On the contrary, if a semiconductor can be tuned to behave as *p* or *n* type, it opens up wider possibilities to design device structure, for example employing homojunctions and there is also a wider choice of partner layers for formation of heterojunctions. Bipolar dopability is therefore desired in the material because it facilitates the optimisation of device design for targeted application.

SnZrSe _3_ is a promising material candidate for photovoltaic application. Nonetheless, to really highlight the potential of this material, the deposition of SnZrSe _3_ in thin film form should be demonstrated. This has not been done or reported thus far. Based on the experience in synthesis of other ABX _3_ chalcogenide thin films (BaZrS _3_) ^16^, deposition process of SnZrSe _3_ could be challenging. Because of the large difference in vapour pressure of constituting elements, conventional chalcogenide thin film synthesis methods can be unsuitable. Therefore, likely alternative synthesis approaches should be explored to synthesize SnZrSe _3_ thin films.

## Conclusions

In this work, we studied the properties of SnZrSe _3_ intending to explore ABX _3_ chalcogenide materials beyond the perovskite structure. We confirmed that the ground phase of SnZrSe _3_ is needle-like (s.g. *Pnma*) where the main building block was a ribbon forming a quasi-one-dimensional crystal structure. Coordination anisotropy around cation A was observed in SnZrSe _3_ which was a sign of a stereochemically active electron lone pair of Sn. The bandgap of SnZrSe _3_ was found to be 1.15 eV which is much smaller than in perovskite chalcogenides, therefore, broadening application range of ABX _3_ chalcogenides. In addition, we found that as-grown SnZrSe _3_ crystals were insulating (ρ _s_ = 10 ^3^-10 ^5^ Ω·cm), showed bipolar dopability and deep intrinsic defects. In terms of ribbon-like crystal structure and optical bandgap, SnZrSe _3_ has similar properties as Sb _2_X _3_ – which is one of the most perspective materials for earth-abundant and non-toxic photovoltaics, but SnZrSe _3_ offers a wider range of tunability in terms of doping and bandgap. However, the next important step in the validation of SnZrSe _3_ prospects is to find a synthesis approach for thin film deposition, which could be not as straightforward as evidenced from experience with perovskite chalcogenides.

## Ethics and consent

Ethical approval and consent were not required.

## Data Availability

Zenodo: Dataset for publication “Synthesis and physical characteristics of narrow bandgap chalcogenide SnZrSe3”. https://doi.org/10.5281/zenodo.7142593 ^22^. This project contains the following underlying data: Sample description.txt (synthesis conditions of the samples presented in the publication). F1IS_V_6244.cif (crystallographic information file about SnZrSe _3_ structure as determined by single-crystal XRD method; CIF can be opened with free of charge available software, e.g. VESTA ( https://jp-minerals.org/vesta/en/), Mercury ( https://www.ccdc.cam.ac.uk/solutions/csd-core/components/mercury/)). XRD.zip (raw XRD patterns of powder samples presented in the publication in .ras and .raw formats; ras file can be read and plotted using open-source platform Labplot; raw/ras files can be opened and analysed in Profex (free of charge, https://www.profex-xrd.org/)). Raman.zip (raw Raman spectra in .txt format and OriginPro project file where data was plotted; alternatively, Raman spectra can also be read and plotted using open-source platform Labplot ( https://labplot.kde.org/)). Optics.zip (raw diffuse reflectance data in .txt format and OriginPro project file where data was processed and plotted; diffuse reflectance spectra can also be read, analysed and plotted using open-source platform Labplot ( https://labplot.kde.org/)). JV-T.zip (I-V curves at specific temperature in .txt format and OriginPro project files where data was processed and plotted; I-V data files can be read, analysed and plotted using open-source platform Labplot ( https://labplot.kde.org/)). Images.zip (optical photographs of the sample and untreated SEM images of crystals). CIF files were deposited with the Cambridge Crystallographic Data Centre CCDC/ICSD, deposition number CSD 2166561, and can be accessed upon request ( https://www.ccdc.cam.ac.uk/structures/). Zenodo: Dataset for publication “Synthesis and physical characteristics of narrow bandgap chalcogenide SnZrSe3”. https://doi.org/10.5281/zenodo.7142593 ^22^. This project contains the following extended data: Extended data.pdf (additional information supporting claims in the publication with direct link to the main text, such as figures). Data are available under the terms of the Creative Commons Attribution 4.0 International license (CC-BY 4.0).
