# Supplementary material for: Synthesis and physical characteristics of narrow bandgap chalcogenide SnZrSe 3
Source: Open Res Eur. 2023 May 17;2:138. Originally published 2022 Dec 13. [Version 2] doi: 10.12688/openreseurope.15168.2 (PMC10445849; doi:10.12688/openreseurope.15168.2)
Supplement: Peer review history

# Peer review: Reviewer response for version 2

**DOI:** 10.21956/openreseurope.17286.r31734

**Published:** 2023-06-01

**Authors:** Hao Zeng

**Affiliations:** 1 Department of Physics, University at Buffalo, Buffalo, NY, USA

I am satisfied with the revised version. The manuscript is ready for indexing.

Is the study design appropriate and does the work have academic merit?

Yes

Is the work clearly and accurately presented and does it cite the current literature?

Yes

If applicable, is the statistical analysis and its interpretation appropriate?

Not applicable

Are all the source data underlying the results available to ensure full reproducibility?

Yes

Are the conclusions drawn adequately supported by the results?

Partly

Are sufficient details of methods and analysis provided to allow replication by others?

Yes

Reviewer Expertise:

Chalcogenide perovskites, optoelectronic properties.

I confirm that I have read this submission and believe that I have an appropriate level of expertise to confirm that it is of an acceptable scientific standard.

# Peer review: Reviewer response for version 1

**DOI:** 10.21956/openreseurope.16407.r31017

**Published:** 2023-03-22

**Authors:** Yi-Yang Sun

**Affiliations:** 1 State Key Laboratory of High Performance Ceramics and Superfine Microstructure, Shanghai Institute of Ceramics, Chinese Academy of Science, Shanghai, China

It is enjoyable to read through this paper, which reports the synthesis and characterization of the compound SnZrSe _3_, an unknown material so far. Even though SnZrSe _3_ appears to be isostrutural to SnZrS _3_, it is an important addition to the database of inorganic crystals. The description on the experimental details and the discussion on the results are detailed and objective enough to assure the reader what has been done and what still requires further study. I have no reservation regarding the indedxing of this work. Nevertheless, given the complete open nature of this journal, I would like to add a discussion which might be of interest to the readers of this paper.

 The "perovskite" structure, without any generalization, should be composed of corner-shared octahedra. In this sense, the ribbon-like structure of SnZrSe _3_ with edge-shared octahedra is not a perovskite. The structure difference will give rise to different electronic structures, which in turn will affect the optical absorption. While this paper measured the optical band gap, the absolute absorption coefficient was not measured. So, whether it can be suitably used for PV is still unclear. The 1.1 eV band gap is already close to the lower bound of the optimal range for a single-junction solar cell. If the fundamental gap is significantly lower than the optical band gap, it will limit the open circuit voltage to a low value.

Is the study design appropriate and does the work have academic merit?

Yes

Is the work clearly and accurately presented and does it cite the current literature?

Yes

If applicable, is the statistical analysis and its interpretation appropriate?

Yes

Are all the source data underlying the results available to ensure full reproducibility?

Yes

Are the conclusions drawn adequately supported by the results?

Yes

Are sufficient details of methods and analysis provided to allow replication by others?

Yes

Reviewer Expertise:

First-principles calculations, Raman spectrum

I confirm that I have read this submission and believe that I have an appropriate level of expertise to confirm that it is of an acceptable scientific standard.

# Response

**Published:** 2023-05-18

**Authors:** Rokas Kondrotas

**Affiliations:** Center for Physical Sciences and Technology, Lithuania

Reviewer #2

 It is enjoyable to read through this paper, which reports the synthesis and characterization of the compound SnZrSe3, an unknown material so far. Even though SnZrSe3 appears to be isostrutural to SnZrS3, it is an important addition to the database of inorganic crystals. The description on the experimental details and the discussion on the results are detailed and objective enough to assure the reader what has been done and what still requires further study. I have no reservation regarding the indexing of this work. Nevertheless, given the complete open nature of this journal, I would like to add a discussion which might be of interest to the readers of this paper.

**Reviewer:** The "perovskite" structure, without any generalization, should be composed of corner-shared octahedra. In this sense, the ribbon-like structure of SnZrSe3 with edge-shared octahedra is not a perovskite. The structure difference will give rise to different electronic structures, which in turn will affect the optical absorption. While this paper measured the optical band gap, the absolute absorption coefficient was not measured. So, whether it can be suitably used for PV is still unclear. The 1.1 eV band gap is already close to the lower bound of the optimal range for a single-junction solar cell. If the fundamental gap is significantly lower than the optical band gap, it will limit the open circuit voltage to a low value.

**Authors:** we thank the reviewer for positive feedback and raising awareness. The ideas that are expressed are very important and deserves a discussion. While providing a response to reviewer #1, some of the concerns have been discussed, and is added in the version 2 of the paper. In particular, the following discussion have been added to account for change in optical properties with respect to crystal structure: On one hand, absorption coefficient (α) depends on the density of states in the bands. If states at direct gap are dispersive it can lead to slowly increasing α resulting in significant absorption onset. Based on optical properties calculated for SnZrSe _3_, α > 104 cm-1 is reached for photon energy > 1.5 eV which renders an onset of around 0.9 eV ^18^. Another theoretical work also shows that among ABX3 chalcogenides those in needle-like phase have a larger absorption onset than those in perovskite structure ^12^, and this was measured for SrZrS _3_ case experimentally [R1]. On the other hand, in materials with dispersive bands, a high carrier mobility can be expected. Consequently, for practical application, for instance solar cells, an optimum absorber thickness will be required to balance between charge carrier mobility, lifetime and absorption coefficient.

 Reference: R1: https://doi.org/10.1002/adma.201604733

# Peer review: Reviewer response for version 1

**DOI:** 10.21956/openreseurope.16407.r30579

**Published:** 2023-01-31

**Authors:** Hao Zeng

**Affiliations:** 1 Department of Physics, University at Buffalo, Buffalo, NY, USA

This paper reports the synthesis of chalcogenide perovskite SnZrSe3, using solid state reaction. Very rarely has selenide perovskite been synthesized and their physical properties characterized. While the method used is not entirely novel, the results will be of great interest to the community working on chalcogenide perovskites. The paper is suitable for indexing after the authors address the following comments:

1. The authors stated that “However, other ABX _3_ chalcogenides although having a non-perovskite ground phase, are also stable compounds, have bandgaps in the infrared region and have high optical absorption.” This is not entirely true. The hexagonal (indirect gap) and need-like phases (pseudo-direct gap) has weaker light absorption than that of the perovskite phase. See Sun’s Nano Lett paper (Ref. 12).
2. In this work, single phase perovskite was not achieved. Instead a mixture of binary phases including ZrSe3, ZrSe2 and SnSe were present. The authors should discuss if there is a pathway towards realizing phase pure materials. Without it the impact of the work is significantly limited.
3. It was shown that when the ampoule was oriented horizontally no secondary Sn-related phases were observed. What happens if excess Sn precursor is used? If there is no secondary phase, what happens to excess Sn?
4. It is stated that first-principles calculations are known to underestimate the bandgap. This is also not true. For example HSE predicted band gaps often are consistent with experimental results and GW sometimes tend to overestimate band gap.
5. It is stated that n-type samples are slightly more Se-rich. However, selenium vacancy should contribute to excess electron carriers. For Se-rich samples, where do excess electrons come from, if not from secondary phase or contaminations? Moreover, how does perovskite accommodate excess Se, unless they occupy cation sites? Such antisite defects are shown to have too high formation energy. Moreover, composition analysis seems to show that the samples are Se deficient (< 60%).
6. Presence of oxygen can lead to apparently higher band gap measured from UV-vis spectroscopy. Can the authors rule out presence of oxygen as the source of their apparently larger band gap than expected?
7. The authors should be careful on claiming presence of both n- and p-type doping, unless they can be sure secondary phases/contaminations are irrelevant. If n- and p-type behavior is indeed attributed to SnZrSe3, defects/dopants should be identified. Furthermore, while authors pointed out that chalcopyrites are often p-type due to Cu vacancies, they failed to mention that both n- and p-type behavior has already been realized in chalcogenide perovskite BaZrS3 using gating. see Yu *et al*, Nano Energy, 85, 105959 ^1^.
8. The authors stated that “SnZrSe _3_ is predicted to have a small (< 0.1 eV) difference between indirect and direct gaps as well. Such characteristic is highly desired in absorber materials for photovoltaics because high absorption coefficient and long carrier lifetime can be realised simultaneously”. Does the theory paper they cited and their own measurement results support this statement? In ref 18, it showed that the onset of absorption is at least .5 eV higher than the predicted band gap. Their own measurements also showed that significant absorption is only available at > 1.5 eV. What is the nature of the direct and indirect gap states? If the direct gap states are dispersive, they will not have high absorption. The authors should put this into perspective by comparing to existing materials (either the calculated dielectric constant or measured absorption coefficient). 

Is the study design appropriate and does the work have academic merit?

Yes

Is the work clearly and accurately presented and does it cite the current literature?

Yes

If applicable, is the statistical analysis and its interpretation appropriate?

Not applicable

Are all the source data underlying the results available to ensure full reproducibility?

Yes

Are the conclusions drawn adequately supported by the results?

Partly

Are sufficient details of methods and analysis provided to allow replication by others?

Yes

Reviewer Expertise:

Chalcogenide perovskites, optoelectronic properties.

I confirm that I have read this submission and believe that I have an appropriate level of expertise to confirm that it is of an acceptable scientific standard, however I have significant reservations, as outlined above.

## References

[1] : Chalcogenide perovskite BaZrS3 thin-film electronic and optoelectronic devices by low temperature processing. *Nano Energy* .2021;85: 10.1016/j.nanoen.2021.105959 10.1016/j.nanoen.2021.105959

# Response

**Published:** 2023-05-18

**Authors:** Rokas Kondrotas

**Affiliations:** Center for Physical Sciences and Technology, Lithuania

Reviewer #1 This paper reports the synthesis of chalcogenide perovskite SnZrSe3, using solid state reaction. Very rarely has selenide perovskite been synthesized and their physical properties characterized. While the method used is not entirely novel, the results will be of great interest to the community working on chalcogenide perovskites.

**Authors**: first of all, we thank reviewers for on point comments and raised questions. In the following report we have addressed these questions as accurate as possible and updated the publication as provided herein.

**Reviewer: **The paper is suitable for indexing after the authors address the following comments:

 The authors stated that “However, other ABX3 chalcogenides although having a non-perovskite ground phase, are also stable compounds, have bandgaps in the infrared region and have high optical absorption.” This is not entirely true. The hexagonal (indirect gap) and needle-like phases (pseudo-direct gap) has weaker light absorption than that of the perovskite phase. See Sun’s Nano Lett paper (Ref. 12).

**Authors:** We do agree with a comment made. To avoid ambiguity and present potential drawbacks having a non-perovskite type ABX _3_ materials, we modified this part of introduction as follows: Other ABX _3_ chalcogenides although in a non-perovskite ground phase have bandgaps spanning from near to mid-wavelength infrared regions ^12^. Additionally, because valence and conduction bands in edge(face)-sharing ABX _3_ chalcogenides is primarily determined by p and d orbitals of X and B elements, respectively, the absorption coefficient is expected to be high as well [R1.1]. Note, however, that lower symmetry crystal structures such as needle-like or BiNiO _3_-type can lead to indirect optical transitions and therefore higher absorption onset as compared with ABX _3_ chalcogenides in perovskite-type structure. Reference: R1.1. https://doi.org/10.1021/acs.inorgchem.8b01038

**Reviewer:** In this work, single phase perovskite was not achieved. Instead a mixture of binary phases including ZrSe3, ZrSe2 and SnSe were present. The authors should discuss if there is a pathway towards realizing phase pure materials. Without it the impact of the work is significantly limited.

**Authors:** when working with this material, indeed we were not able to achieve high yield of SnZrSe _3_ phase. Traditionally, in a solid-state synthesis one focuses on finding optimal temperature and ensuring that precursors are evenly distributed through regrinding and mixing steps. Meanwhile duration of the annealing process was not taken into account having a perception that the longer process - the better. While continuing our work on the synthesis optimisation we noticed an interesting feature. Samples that were annealed for a shorter duration had higher yield of SnZrSe _3_ phase. So we made additional experiments where only duration process was varied and found that shorter durations than we typically used for our process yielded a higher concentration of SnZrSe _3_ phase. Having these new insights in the experiment we have updated the publication as follows: *-Slightly corrected the experimental section to include new annealing parameters:* Ampoules were introduced in the tube furnace, 13 of them in a horizontal and 3 of them in a vertical (placed in ceramic holder) positions at the centre of the heating zone. The heating process included either one or two steps: (i) temperature was raised to the top temperature within 3 – 7 h which was varied in the 600 – 800 °C range and held for 10 – 80 h; (ii) in the second step, the temperature was reduced to 100 – 600 °C within 7 – 60 h, and furnace power was switched off leaving to cool down naturally for a few hours. We found that larger crystals were formed when the temperature was reduced slowly in the second stage, instead of maintaining a high furnace temperature for a prolonged period of time. Note: initially we presented that annealing at top temperature was held for 10 – 80 h, which suggests that we tested short annealing durations. However, a short duration was used in two-step annealing process where in the second step temperature was slowly (~0.03 ^o^C/min) reduced. Therefore, technically duration at the crystallization temperature range was 60 – 80 h. *-Rewritten first paragraph about solid state synthesis as follows:* SnZrSe _3_ was synthesized via solid state reaction from precursors in elemental form. Unless stated otherwise, a transport agent (SnI _2_) was used to accelerate the reaction and to grow single crystals. In other cases, no additives were introduced. In most of experiments, various shapes, sizes and forms of crystals were present inside an ampoule after annealing indicating formation of secondary phases. By analysing XRD data we found that in addition to SnZrSe _3_ phase, one or two of the following binary phases were present: ZrSe _3_ (ICDD# 03-065-2351), ZrSe _2_ (ICDD# 04-005-5128) and SnSe (ICDD# 04-009-2257). Based on multiple trials, we noticed that the following experimental parameters had a deciding factor on the phase composition: (i) spatial separation of precursor materials (ii) Se overpressure in the ampoule and (iii) annealing duration. When the ampoule was oriented vertically in the annealing zone, ZrSe _3_ and traces of SnSe phases were present despite the annealing temperature applied ( Figure 1a). Interestingly, the addition of transport agent in a form of SnI _2_ did not change the phase composition. This suggested that when precursors were in close proximity, the solid-state reaction was not accelerated or favoured for the formation of SnZrSe _3_ in the presence of a transport agent. When ampoules were oriented horizontally and precursor powder was spread over a certain length, phase composition strongly depended on the initial Se content. Using Se-deficient precursor, a large amount of ZrSe _2_ and absence of ZrSe _3_ phases were observed in the powder ( Figure 1b). Once stoichiometric or Se-rich precursors were used, little to no amount of ZrSe _2_ was found, which indicated that the formation of ZrSe _3_ phase was highly favoured under Se-rich atmosphere. Note that when the ampoules were oriented horizontally no secondary Sn-related phases were observed. Finally, the annealing duration at the top temperature had a significant effect on SnZrSe _3_ powder phase purity. In the initial experiments we used long annealing durations to facilitate a complete solid-state reaction. However, we noticed that shorter annealing durations yielded lower concentration of secondary phases (Figure 1 c). In the samples annealed under identical conditions but with different durations, we found that almost pure-phase powder was obtained annealing for 35 h instead of 60 h or more used previously ( Figure 1c ). Longer or shorter than 35 h annealing durations resulted in the formation of ZrSe _2_ phase which concentration was much higher in the sample synthesized for a long period of time. This suggested that SnZrSe _3_ formed relatively quickly and under prolonged annealing time slowly decomposed into binary phases. Therefore, from these results we can infer that 700 ^o^C is close to the decomposition temperature of SnZrSe _3_. *- Modified final paragraph about solid-state:* Therefore, we believe that the thermodynamic balance between reactions (3) and (4) which depends on spatial separation of materials, partial Se pressure and annealing temperature determine the final product composition. While inspecting the ampoule annealed sequentially first at 700 °C and then at 800 °C, it was noticed that after the second annealing large SnZrSe _3_ crystals deteriorated, and small fine needle-like crystals typical for ZrSe _3_ were found (Figure S1 in Extended data ^22^). This indicated that SnZrSe _3_ decomposes at higher temperatures. This observation was in agreement with the results obtained from time-dependent annealing series, where prolonged synthesis duration at 700 °C led to decomposition of SnZrSe _3_. Therefore, to synthesize SnZrSe _3_ phase with high yield, it is essential to keep balance between top temperature and annealing duration. With our experimental setup it was around 35 h at 700 °C. However, if lower temperature is applied, longer duration may be required and vice versa.

*Updated Figure 1 by adding powder patterns of samples synthesized for different annealing durations: *Figure 1. (a) Diffractograms of SnZrSe _3_ powder synthesized at various temperatures in a vertical position without and with transport agent. (b) Diffractograms of SnZrSe _3_ powder samples synthesized in the same experiment at 740 °C temperature in a horizontal position with different initial Se-content in the precursor as indicated in the graph. ( c) Diffractograms of SnZrSe _3_ powder annealed for different durations. Top temperature – 700 °C, SnI _2_ transport agent, position – horizontal, composition - stoichiometric. (d) XRD pattern of collected and grinded crystals synthesized at 700 °C in horizontal position with a transport agent. Diffractogram of pure SnZrSe _3_ phase was simulated using CIF file (underlying data ^22 ^).

**Reviewer:** In It was shown that when the ampoule was oriented horizontally no secondary Sn-related phases were observed. What happens if excess Sn precursor is used? If there is no secondary phase, what happens to excess Sn?

**Authors:** that is a valid point which we were asking ourselves too. Naturally, if we have a decomposition or incomplete reaction of binary phases we should be able to observe both Zr-related and Sn-related secondary phases. This, however, was not always the case. We are not entirely certain about the mechanism, but a current hypothesis is as follows: Sn-related phases were not observed in samples when horizontal configuration was used or higher than 700 ^o^C temperature applied. In these ampoules, at the very end of ampoule (photo below), optically we observed condensation of material which had a mixture of metallic and red lustre. Usually, this part material was not collected for analysis because of difficulty to scratch it off from uneven quartz ends and was assumed to be condensate of transport agent. However, in few samples we have carried out chemical analysis of these droplets and found elements Sn, Se and I. This suggested that not only transport agent but also some Sn-Se phase condensed on ampoules ends. At high temperature (> 700 ^o^C) all SnSe whether as a product of decomposition or incomplete reaction is likely in vapour phase and with a help of iodine is transferred to the coldest part of ampoule. Very likely this occurred during cool down step. So our primary explanation is that excess of Sn condensed on the very ends of ampoules and was not collected ( Figure 2).

**Reviewer:** It is stated that first-principles calculations are known to underestimate the bandgap. This is also not true. For example HSE predicted band gaps often are consistent with experimental results and GW sometimes tend to overestimate band gap.

**Authors: **we do agree with reviewer’s comment on the first principal high accuracy in predicting bandgap using hybrid functionals such as HSE06. The discrepancy we found in experimentally measured bandgap and the one calculated using mBJ (potential that also produces bandgaps consistent with experiments) was quite large i. e. 1.1 eV (exp) vs 0.53 eV (calc). We could argue that our experimental way of defining bandgap (using diffuse reflectance) could potentially not account for weak indirect optical transitions, and that real indirect bandgap would be within error of calculated value. But based on the band structure the difference between direct and indirect gaps in SnZrSe _3_ and SnZrS _3_ is < 0.1 eV [T7]. This therefore cannot account for a large difference (> 0.5 eV) between experimental and calculated values. Note that this discrepancy is more pronounced for needle-like ABX _3_ chalcogenides as shown in the Table 1 below. Based on this we conclude that needle-like phase ABX _3_ chalcogenides are less accurately described in DFT than perovskite or hexagonal phases. Additionally, in SnZrSe _3_ there exist a lone electron pair of Sn, which can further complicate high accuracy simulations using DFT.

 Table 1. Bandgaps of selected ABX _3_ chalcogenides determined experimentally and calculated using first principals. Compound Structure Exp E _g_, eV Calc* E _g_, eV ΔEg, eV Ref. SrZrS _3_ DP 2.13 1.95 0.18 [T1] SrZrS _3_ **N** 1.53 1.17 **0.36** [T1] BaZrS _3_ DP 1.81 1.75 0.06 [T1] BaTiS _3_ H 0.27** 0.16 0.11 [T2] SrTiS _3_ H 0.25** 0.25 0.0 [T3] SrHfS _3_ DP 2.32 2.28 0.06 [T4] BaHfS _3_ DP 2.06 2.0 0.06 [T4] SrHfSe _3_ **N** 1.02 0.79 **0.24** [T5] SnZrSe _3_ **N** 1.10 0.63 **0.47** This work DP – distorted perovskite structure, prototype GdFeO _3_ N – needle-like structure, prototype NH _4_CdCl _3_ H – hexagonal structure, prototype BaNiO _3_ * - all calculated values are taken from the work of Sun et al. [T6], except for SnZrSe _3_ [T7]. Reference is provided for experimentally determined bandgap values. ** - absorption edge.

 Taking the comments into account, we have modified the discussion part regarding calculated and observed bandgaps as follows: First-principles calculations using hybrid potentials can predict bandgaps consistent with experimental values therefore such large discrepancy we relate to one of the two reasons: (i) SnZrSe _3_ is an indirect bandgap semiconductor and using diffuse reflectance method we were not able to clearly detect weak indirect optical transitions which would be expected to be lower in energy than direct (ii) due to low density of states at the band edges, strong optical absorption starts with an high onset leading significant absorption only at higher photon energies ^18^.

 References: T1. https://doi.org/10.1002/adma.201604733 T2. https://doi.org/10.1038/s41566-018-0189-1 T3. https://doi.org/10.1021/acs.chemmater.8b02279 T4. https://doi.org/10.1021/jacs.8b13622 T5. https://doi.org/10.1021/acs.inorgchem.8b01038 T6. https://doi.org/10.1021/nl504046x T7. https://doi.org/10.1016/j.mtcomm.2021.102427

**Reviewer:** It is stated that n-type samples are slightly more Se-rich. However, selenium vacancy should contribute to excess electron carriers. For Se-rich samples, where do excess electrons come from, if not from secondary phase or contaminations? Moreover, how does perovskite accommodate excess Se, unless they occupy cation sites? Such antisite defects are shown to have too high formation energy. Moreover, composition analysis seems to show that the samples are Se deficient (< 60%).

**Authors: **first, we would like to clarify that when we used the term “more Se-rich” we meant Se content when comparing samples with each other, but not with expected ABX _3_ stoichiometric composition 1:1:3. We have clarified this as follows: In both samples, the cationic ratio A/B was identical, whereas the n-type crystal was slightly more Se-rich than p-type, but both were Se-deficient with respect to stoichiometric ratio of [X]/([A]+[B]) which is 1.5. All samples which we have analysed can be considered as Se-deficient and cation-rich with respect to an ideal 1:1:3 ratio. In terms of atomic percentage of Se it was always found to be in 57 – 58.5 at.% range. If high oxygen contamination was present, even lower Se concentration was found. However, even for very clean as-grown crystals measured immediately after synthesis where no oxygen was present, the Se content was no more than 58.5 at.%. There are two possible reasons for this observation: (i) deviation could be related to the high measurement system error ( > 2 at.%). Although this is unlikely because using the same conditions we measured the composition of GaAs, GeS, GeSe monocrystals on the same system and all them showed a 50:50 atomic ratio with 0.5 at.% error. Another reason could be that a equilibrium composition of SnZrSe _3_ is not 1:1:3, but slightly cation-rich and anion-deficient such as: 1.05 : 1.05 : 2.9. In fact, other ABX _3_ chalcogenides in needle-like or hexagonal phases were also found not to follow 1:1:3 ratio strictly, e. g. Ba _1.12_Zr _1.0_Se _3.01_, Sr _21_Ti _19_Se _57_ [R5.1] or Sn _1.2_Ti _.08_S _3_ [R5.2] Ba _1.06_Ti _1_S _2.95_ [R5.3]. Thus it is not known for certain what is the precise equilibrium stoichiometry of SnZrSe _3_ compound and at which point it can be regarded as Se-rich or Se-poor. Therefore, in our work we focused on comparing and finding trends within sample series rather than with absolute values. Additionally, we have strong evidence that cations play more important role than anion in defect chemistry related to conductivity on which we elaborated more in question 7. Because of the reasons outlined above, the discussion about Se-vacancy defects as potential donors becomes speculative. Although we cannot completely rule out the contribution of V _Se_, there is a stronger correlation of conductivity type and carrier concentration with cation composition.

 References: R5.1. https://doi.org/10.1021/ja972442p R5.2. https://doi.org/10.1063/1.5093183 R5.3. https://doi.org/10.1038/s41566-018-0189-1 (in supporting information)

**Reviewer:** Presence of oxygen can lead to apparently higher band gap measured from UV-vis spectroscopy. Can the authors rule out presence of oxygen as the source of their apparently larger band gap than expected?

**Authors: **we agree that oxygen if incorporated in the lattice and resulting in formation of SnZr(Se,O) _3_ alloy would lead to higher than expected bandgap as was observed in [R6.1]. However, there are two reasons why we think oxygen had no contribution in optical absorption in our case: (i) first powder sample that were used to measure bandgap was obtained by grinding needle-shaped crystals. Compositional analysis of the crystals usually showed no content or very minor traces of oxygen. Higher content of oxygen was observed when crystals were grounded to powder and exposed for prolonged time under ambient. This shows that during synthesis process there was no contamination with oxygen, and it was not incorporated in the lattice. It is very unlikely that at room temperature oxygen from air would be incorporated into the crystal lattice of SnZrSe _3_ and lead to formation of SnZr(Se,O) _3_. (ii) Another evidence that oxygen does not enter SnZrSe _3_ lattice is based on the lattice parameters of SnZrSe _3_. If oxygen was incorporated in the crystal lattice, the lattice parameters of SnZrSe _3_ would shrink because of much smaller ionic radius of O than Se. In the following Table 2 we present lattice parameters calculated for one of our SnZrSe _3_ powder:

 Table 2. Lattice constants of SnZrSe _3_ Sample a, Å b, Å c, Å As synthesized 9.5940 3.8463 14.4087 Aged 7 months 9.5936 3.8462 14.4082 Single crystal 9.5862 3.8443 14.3959

 It is quite clear that lattice constants do not change with time indicating there is no change in SnZrSe _3_ lattice under ambient conditions, and that lattice parameters calculated for powder sample is slightly higher than measured in single crystal. If oxygen was incorporated in the lattice, there would be a much more significant shift of lattice constants and toward smaller not higher values. Reference: R6.1. https://doi.org/10.1021/acs.jpclett.1c00177

**Reviewer:** The authors should be careful on claiming presence of both n- and p-type doping, unless they can be sure secondary phases/contaminations are irrelevant. If n- and p-type behavior is indeed attributed to SnZrSe3, defects/dopants should be identified. Furthermore, while authors pointed out that chalcopyrites are often p-type due to Cu vacancies, they failed to mention that both n- and p-type behavior has already been realized in chalcogenide perovskite BaZrS3 using gating. see Yu et al, Nano Energy, 85, 1059591.

**Authors: **we do agree that claims about n- and p-type doping engineering in SnZrSe _3_ based on intrinsic defects lack more evidence therefore we acknowledged a possible contribution of impurities in the following part: **“**Important to note that since iodine was used as a transport agent to facilitate the solid state reaction during synthesis, it could be inadvertently introduced in the lattice giving rise to extrinsic doping. Iodine could not be detected by EDX, but we acknowledge that very small amounts (beyond the EDX detection limit) can have a significant contribution to the conductivity behaviour.” Because single crystals were used for electrical measurements, we are quite certain that there is no contribution to conductivity from secondary phases. Secondly, because we were able to observe p-type conductivity, we can be sure, that p-type can be achieved to some extent by modifying SnZrSe _3_ composition alone. Finally, although extrinsic doping via iodine impurities is possible, in chalcogenides doping of halogens usually introduce shallow donor defects, e.g. ZnSe:I E _a_ = 0.026 eV [R7.2], SnS:Cl E _a_=0.0015 eV [R7.3], Sb _2_Se _3_:Br E _a_=0.06 eV (calculated) [R7.4]. Therefore, despite whichever sample we test, we should be able to observe additional probably low activation energy in the conductivity vs temperature graph. This was not the case. So we strongly believe that n- and p-type doping originated from intrinsic SnZrSe _3_ defects. To identify which point defects are responsible for generating free electrons/holes is very difficult without knowledge of defect chemistry in SnZrSe _3_ on theoretical basis. This is our initial work on SnZrSe _3_ and in the future it would be extremely useful to analyse defect chemistry from first-principles. This then would allow us to identify potential free carrier generating defects. Additionally, currently we are working of the synthesis of Sn(Zr _1-x_Ti _x_)Se _3_ alloy system and observed a very strong correlation between change of resistivity (5 orders of magnitude) and Ti content which again points to the fact that cations rather than anion or extrinsic doping is more critical to electrical behaviour of SnZrSe _3_. Regarding the achievements in observing both type carriers in BaZrS _3_, this is indeed an important milestone and this publication was added in the main text: In this work, we found that SnZrSe _3_ showed *p* as well as *n* type conductivity behaviour. Ambipolar behaviour was also recently realised in BaZrS _3_ thin films [R7.1].

 Reference R7.1. https://doi.org/10.1016/j.nanoen.2021.105959 R7.2. https://doi.org/10.1016/0022-0248(88)90607-0 R7.3. https://doi.org/10.1021/acs.cgd.0c00617 R7.4. https://doi.org/10.1021/acsaem.9b02192

**Reviewer:** The authors stated that “SnZrSe3 is predicted to have a small (< 0.1 eV) difference between indirect and direct gaps as well. Such characteristic is highly desired in absorber materials for photovoltaics because high absorption coefficient and long carrier lifetime can be realised simultaneously”. Does the theory paper they cited and their own measurement results support this statement? In ref 18, it showed that the onset of absorption is at least .5 eV higher than the predicted band gap. Their own measurements also showed that significant absorption is only available at > 1.5 eV. What is the nature of the direct and indirect gap states? If the direct gap states are dispersive, they will not have high absorption. The authors should put this into perspective by comparing to existing materials (either the calculated dielectric constant or measured absorption coefficient).

**Authors:** Regarding the first question, we do not have estimates of carrier lifetime therefore we cannot fully support the statement. Whether bands are dispersive we can only rely on ref 18 results, which suggest that it strongly depends on the crystallographic direction. Near the indirect gap region bands are indeed flat which would suggest high optical absorption, whereas in other directions they are dispersive, e.g. Γ→ R (figure below). Note that in ref 18 only the calculated values of absorption coefficient are presented. However, we do acknowledge that larger absorption onset was observed in ABX _3_ chalcogenides of needle-like phase and therefore extent the discussion in the manuscript as follows: On one hand, absorption coefficient (α) depends on the density of states in the bands. If states at direct gap are dispersive it can lead to slowly increasing α resulting in significant absorption onset. Based on optical properties calculated for SnZrSe _3_, α > 10 ^4^ cm ^-1^ is reached for photon energy > 1.5 eV which renders an onset of around 0.9 eV ^18^. Another theoretical work also shows that among ABX _3_ chalcogenides those in needle-like phase have a larger absorption onset than those in perovskite structure ^12^, and this was measured for SrZrS _3_ case experimentally [R8.1]. On the other hand, in materials with dispersive bands, a high carrier mobility can be expected. Consequently, for practical application, for instance solar cells, an optimum absorber thickness will be required to balance between charge carrier mobility, lifetime and absorption coefficient.

 Electronic band dispersion curves of SnZrSe _3_ along high symmetry direction of the Brillouin zone. Adapted from ref.18. Reference: R8.1: https://doi.org/10.1002/adma.201604733
